# A Multidisciplinary Approach and Current Perspective of Nonalcoholic Fatty Liver Disease: A Systematic Review

**DOI:** 10.7759/cureus.29657

**Published:** 2022-09-27

**Authors:** Chowdhury F Zaman, Jakia Sultana, Proma Dey, Jui Dutta, Sadia Mustarin, Nuzhat Tamanna, Aditi Roy, Nisha Bhowmick, Mousumi Khanam, Sadia Sultana, Selia Chowdhury, Farjana Khanam, Md Sakibuzzaman, Priyata Dutta

**Affiliations:** 1 Medicine and Surgery, Jahurul Islam Medical College and Hospital, Kishoreganj, BGD; 2 Medicine, Comilla Medical College, Cumilla, BGD; 3 Internal Medicine, Chittagong Medical College, Chattogram, BGD; 4 Medicine, Mymensingh Medical College, Mymensingh, BGD; 5 Medicine, Rangpur Medical College and Hospital, Rangpur, BGD; 6 Medicine, Sher-e-Bangla Medical College, Barisal, BGD; 7 Medicine, Shaheed Ziaur Rahman Medical College, Bogra, BGD; 8 Internal Medicine, Dhaka Medical College, Dhaka, BGD; 9 Medicine, Anwer Khan Modern Medical College, Dhaka, BGD; 10 Medicine, Central Medical College, Cumilla, BGD; 11 Neurology, The University of Toledo, Toledo, USA; 12 Internal Medicine, University of Mississippi Medical Center, Jackson, USA; 13 Internal Medicine, Sir Salimullah Medical College, Dhaka, BGD; 14 Experimental Pathology (Cancer Biology), Mayo Clinic, Rochester, USA; 15 Internal Medicine, Trinity Health, St. Joseph Mercy Ann Arbor, Ann Arbor, USA

**Keywords:** nafld, nonalcoholic fatty liver disease, covid-19, gut microbiota, hepatocellular carcinoma (hcc), metabolic syndrome, obesity, nonalcoholic fatty liver disease (nafld)

## Abstract

In recent times, nonalcoholic fatty liver disease (NAFLD) has been considered one of the major causes of liver disease across the world. NAFLD is defined as the deposition of triglycerides in the liver and is associated with obesity and metabolic syndrome. Hyperinsulinemia, insulin resistance (IR), fatty liver, hepatocyte injury, unbalanced proinflammatory cytokines, mitochondrial dysfunction, oxidative stress, liver inflammation, and fibrosis are the main pathogenesis in NAFLD. Recent studies suggest that the action of intestinal microbiota through chronic inflammation, increased intestinal permeability, and energy uptake plays a vital role in NAFLD. Moreover, polycystic ovarian syndrome also causes NAFLD development through IR. Age, gender, race, ethnicity, sleep, diet, sedentary lifestyle, and genetic and epigenetic pathways are some contributing factors of NAFLD that can exacerbate the risk of liver cirrhosis and hepatocellular carcinoma (HCC) and eventually lead to death. NAFLD has various presentations, including fatigue, unexplained weight loss, bloating, upper abdominal pain, decreased appetite, headache, anxiety, poor sleep, increased thirst, palpitation, and a feeling of warmth. Some studies have shown that NAFLD with severe coronavirus disease 2019 (COVID-19) has poor outcomes. The gold standard for NAFLD diagnosis is liver biopsy. Other diagnostic tools are imaging tests, serum biomarkers, microbiota markers, and tests for extrahepatic complications. There are no specific treatments for NAFLD. Therefore, the main concern for NAFLD is treating the comorbid conditions such as anti-diabetic agents for type 2 diabetes mellitus, statins to reduce HCC progression, antioxidants to prevent hepatocellular damage, and bariatric surgery for patients with a BMI of >40 kg/m^2^ and >35 kg/m^2^ with comorbidities. Lifestyle and dietary changes are considered preventive strategies against NAFLD advancement. Inadequate treatment of NAFLD further leads to cardiac consequences, sleep apnea, chronic kidney disease, and inflammatory bowel disease. In this systematic review, we have briefly discussed the risk factors, pathogenesis, clinical features, and numerous consequences of NAFLD. We have also reviewed various guidelines for NAFLD diagnosis along with existing therapeutic strategies for the management and prevention of the disease.

## Introduction and background

Nonalcoholic fatty liver disease (NAFLD) comprises a wide spectrum of lesions ranging from benign simple steatosis to nonalcoholic steatohepatitis (NASH) and more advanced diseases, including fibrosis and cirrhosis [1-3]. In the global population, the prevalence of NAFLD is approximately 25% [4], whereas, for NASH, it is 3% to 5% [4].

NAFLD is considered the hepatic manifestation of systemic insulin resistance associated with obesity, type 2 diabetes mellitus (T2DM), and metabolic syndrome [5-7]. Though obesity and metabolic syndrome are the most important risk factors for NAFLD [4], there are behavioral, environmental, and/or genetic risk factors that alter the progression of the disease [2]. High carbohydrate and fat intake, hyperinsulinemia, and subsequently insulin resistance cause the buildup of fatty acids (FA) and triglycerides (TGs) in the liver by decreasing very low-density lipoprotein (VLDL) secretion and the transfer of free fatty acids (FFA) from the liver [8]. FFA further triggers lipotoxicity and leads to activation of fibrogenesis and liver disease [5]. Additional factors that may be responsible for the progression of NAFLD are changes in gut microbiota composition, the function of the gut barrier, and microbial metabolism [2]. NAFLD is a multisystemic disease with extrahepatic complications such as cardiovascular disease [9], chronic kidney disease (CKD) [5,10,11], diabetes mellitus [10,11], extrahepatic cancer such as colon cancer, esophageal cancer, cholangiocarcinoma [10,11], polycystic ovarian disease [12], and inflammatory bowel disease (IBD) [13]. Among them, cardiovascular disease is the leading cause of mortality in NAFLD patients [5,14,15]. The ongoing coronavirus disease 2019 (COVID-19) pandemic raised concern due to its effect on NAFLD patients. Although the clinical features of COVID-19 patients with NAFLD are not clearly understood [16], it has been seen that NAFLD patients with pre-existing obesity and metabolic syndrome suffer from a severe form of COVID-19 [17].

Among the current diagnostic tests available, liver biopsy is considered the key tool to diagnose NAFLD [6]. The NASH test diagnoses and categorizes NASH into three groups, namely, NASH, borderline NASH, and no NASH [18]. Patients with NAFLD with fibrosis can be identified with accuracy using a set of microbial indicators coupled with age and BMI [19].

There are various interventions that can prevent NAFLD-related complications. Among them, lifestyle changes with weight loss may improve steatosis, inflammation, and even fibrosis [20]. A promising dietary intervention is the Mediterranean diet [21]. Other than that, pharmacotherapy, including metformin, liraglutide, and statin, has been demonstrated to reduce steatosis in those with NASH and prevent liver events in patients with metabolic syndrome with advanced NASH [21]. While in the existing articles, there is a dearth of detailed overviews of NAFLD, our article provides a comprehensive idea, from risk factors to current treatment of NAFLD. This systematic review gives us the opportunity to get a prompt overview of the current perspective of global prevalence, pathophysiology, preventive approach, early diagnosis, and the current treatment regimen of this emerging disease.

## Review

Methodology

**Figure 1:**
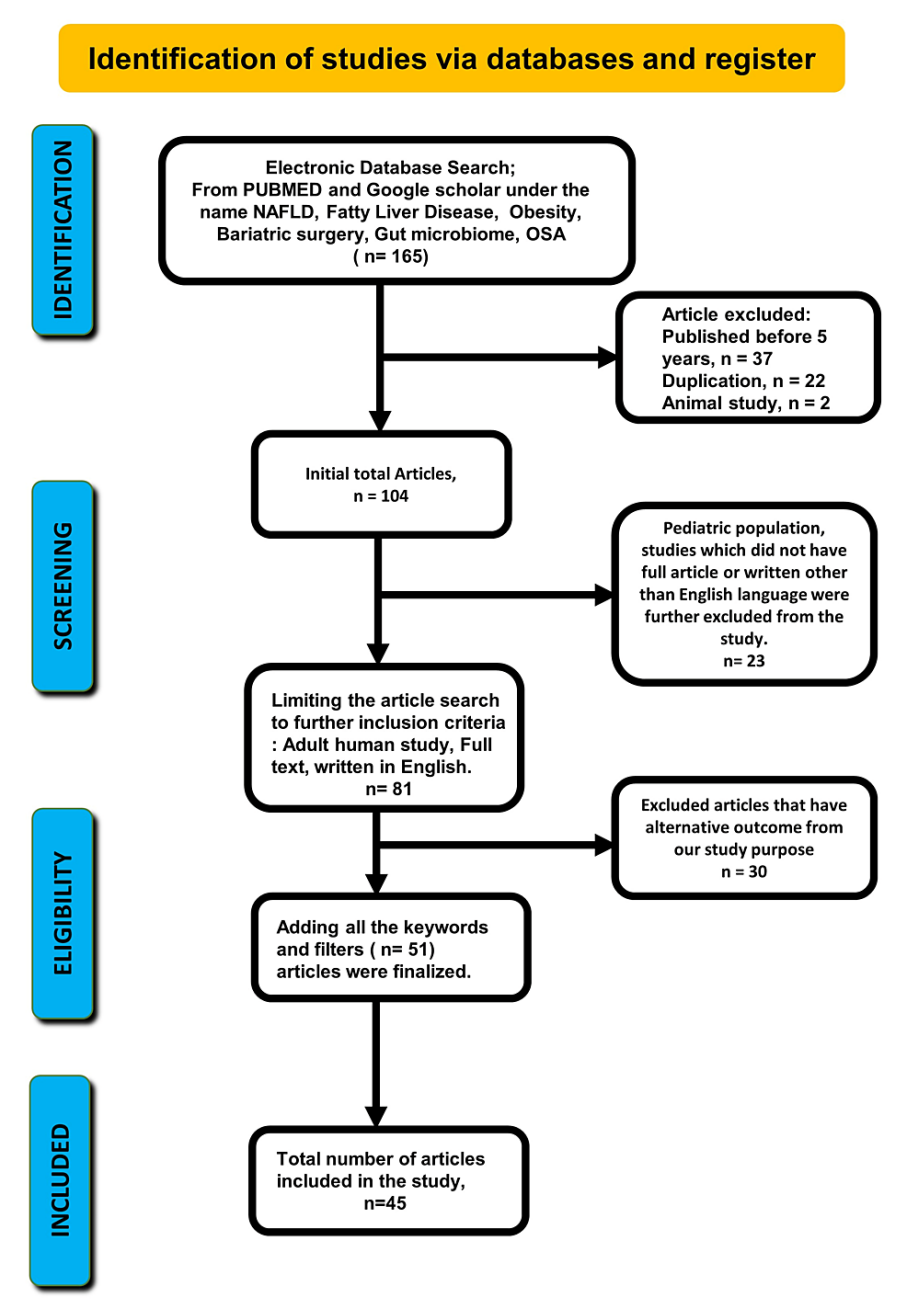
Preferred Reporting Items for Systematic Reviews and Meta-Analyses (PRISMA) flow diagram shows the study selection process. OSA = obstructive sleep apnea; NAFLD = nonalcoholic fatty liver disease.

We searched for studies in PubMed and Google Scholar using the following keywords: “NAFLD,” “fatty liver disease,” “obesity,” “diabetes,” “bariatric surgery,” “obstructive sleep apnea (OSA),” and “gut microbiome.” Initially, 115 articles were collected from PubMed and 50 articles were collected from Google Scholar (Figure 1). The Preferred Reporting Items for Systematic Reviews and Meta-Analyses (PRISMA) 2020 guidelines were followed during the screening phase to prevent duplication [22]. Furthermore, the included studies were screened with the following inclusion criteria: studies published within the last five years (2017-2021), on adult (18 years or more) human subjects, written in English, and available in full articles. Our exclusion criteria were articles published before 2017, case reports, articles that included the pediatric population, animal studies, and articles written in languages other than English. Studies that did not meet our inclusion criteria were excluded. And the total number of articles was 81. Articles with a poor design or an alternative outcome from our study purpose were further excluded. A total of 51 articles were chosen after evaluating and modifying the search results. From there, 45 review articles had the most necessary information, so we finally used those 45 articles in our study.

Discussion

The incidence of NAFLD is 15-30% in the general population [8]. The prevalence of NAFLD has increased among children and the elderly in many nations. Studies have shown that 25% of adults in the USA and one-third of individuals in Japan were found to have NAFLD annually [2]. Moreover, in China, the rate of fatty liver disease is increasing at a rate of 0.594% per year [2]. In Israel, the incidence rate of NAFLD is reported to be 0.028% [23]. Based on a meta-analysis, the global prevalence of NAFLD in adults is approximately 25.24% [23]. The highest prevalence of NAFLD is found to be in the Middle East (31.79%) and South America (30.45%), while the lowest prevalence is shown to be in Africa (13.48%) [23]. In Iran, the prevalence of NAFLD and NASH varies from 2.9% to 7.1% in the general population [24].

Risk Factors

Age and gender: The prevalence of NAFLD is 34% in people aged more than 70 years, whereas 22.4% are between 30 and 39 years old [25]. According to a population-based study that was done in Hong Kong and China, the prevalence of NAFLD is >50% in patients who are older than 60 years [25]. NAFLD prevalence is shown to be higher in men than in premenopausal women [26].

Family history and genetics: Steatosis level is increased in people who are homozygous for the I148M variant of the patatin-like phospholipase domain-containing protein 3 (PNPLA3) allele compared to those who are not carriers of this [27]. They show a 30-50% greater chance of progression from NAFLD to fibrosis and cirrhosis [27]. The risk of NAFLD progression to advanced disease is 18% in patients with first-degree relatives with NAFLD, whereas 1% with no family history [3].

Diet: Patients with NAFLD are shown to consume about two to three times more fructose than those without the disease [28]. NAFLD also is related to high fat intake, especially saturated and polyunsaturated FA [29,30]. Dietary data show that about 14% of the total energy of NAFLD patients is from consumed saturated fat, whereas it is 10% in the non-diseased group [1].

Obesity and metabolic syndrome: Among NAFLD patients, about 75% of patients are overweight, and 90-95% are morbidly obese [31]. Liver biopsy findings of NAFLD patients correlated with the severity of obesity in about 90% of cases [32]. Around 98% of obese patients have hepatic steatosis on ultrasound (US) findings [5]. The amount of liver fat is remarkably higher in patients with metabolic syndrome compared to the general population [29]. In patients with T2DM, the incidence of NAFLD is around 75% [29]. According to a meta-analysis of 86 studies, different associated factors are found in NAFLD patients, which are given in Table 1 [28].

**Table 1:** Associated factors (in %) related to NAFLD. Adapted from [28]. NAFLD = nonalcoholic fatty liver disease.

Associated factor	Percentages found in NAFLD patients
Obesity	51.3%
Type 2 diabetes mellitus	22.5%
Hyperlipidemia	69.2%
Hypertension	39.3%

Diagnostic Criteria

NAFLD is characterized by the presence of excessive accumulation of hepatic fat in liver parenchyma determined by histology or imaging and ruling out other secondary causes like alcohol consumption, medications, or hereditary disorder [23]. NAFLD is differentiated from NASH without lobular inflammation or hepatocyte ballooning degeneration [33]. The diagnosis criteria for NAFLD vary based on various guidelines as given below [33].

According to the European Association for the Study of the Liver (EASL), the diagnosis criteria for NAFLD are as follows: steatosis in 5% of hepatocytes by either imaging or histology and no other causes of steatosis, as well as insulin resistance (IR) and alcohol consumption thresholds for men and women at <30 g/d and 20 g/d, respectively [33].

The guidelines from the National Institute for Health and Care Excellence (NICE) state excessive fat in the liver with no other causes of steatosis and impose restrictions on alcohol consumption where the threshold for men and women is 30 g/d and 20 g/d, respectively [33].

Besides, the guideline developed by Asia-Pacific focuses on hepatic steatosis by either imaging or histology and no other causes of steatosis; alcohol consumption restrictions are two standard drinks/day or 140 g/week for men and one standard drink/day or 70 g/week for women [33].

Both the Italian Association for the Study of the Liver (AISF) and the American Association for the Study of Liver Diseases (AASLD) guidelines are similar, which state that hepatic steatosis can be diagnosed by imaging or histology in the absence of any other causes of steatosis. Besides, alcohol consumption per AISF threshold is <30 g/d and 20 g/d for men and women, respectively. On the other hand, as per AASLD guidelines, the alcohol consumption threshold is 21 standard drinks/week or 294 g/week for men and 14 standard drinks/week or 196 g/week for women [33].

Clinical Features

The clinical presentation of NAFLD is widely variable. Most cases remain asymptomatic until the later stages of illness [2,20,29,34]. Asymptomatic patients are diagnosed with abnormal liver function tests or imaging showing hepatic steatosis when they come for routine examination or follow-up care requiring measurement of liver enzymes [2,20,24,29]. Symptomatic patients present with many ubiquitous symptoms such as fatigue, malaise, unexplained weight loss, right upper abdominal discomfort, and anxiety [20,24,29,34]. In a study, a wide array of presentations such as upper abdominal pain, decreased appetite, morning heaviness, anxiety, headache, bloating, poor sleep quality, late-onset sleeping, feeling of warmth, palpitation, and increased thirst were discovered to be the prevalent clinical manifestations [24].

Relation With Genetic and Environmental Factors

Both genetic and environmental factors contribute to the development of NAFLD. Obesity leads to IR and hyperinsulinemia, the primary determinant of environmental factors. IR and hyperinsulinemia alter the phospholipids and TGs structure by the hydrolysis of oleic acid [3]. The PNPLA3 protein, located at the top of lipid droplets, is activated by insulin [3]. In the case of IR, I148M protein, a variant of PNPLA3, undergoes a missense mutation, leading to the substitution of isoleucine to methionine at position 148. This mutated I148M protein later accumulates on the surface of lipid droplets, causing NAFLD and steatosis [27]. Other genetic forms, such as APOC3, GCKR, TM6SF2, PPP1R3B, LYPLAL1, and NCAN, also contribute to NAFLD [25].

Relation With Metabolic Factor

There lies a "two-hit theory" in the pathogenesis of NAFLD development [1]. The appearance of metabolic syndrome and TG aggregation in the liver is described as the first-hit theory [1]. High dietary carbohydrate and fat intake causes metabolic syndrome, which consists of central obesity, elevated blood pressure, dyslipidemia (decreased high-density lipoprotein cholesterol, increased low-density lipoprotein cholesterol, and TG in the blood), hyperglycemia, and IR [30]. IR and hyperinsulinemia further cause the buildup of FA in the liver by decreasing VLDL secretion and the transfer of FFA from the liver [8]. A carbohydrate (CHO) diet, consisting of monosaccharides like glucose and sucrose, also turns into FA through the liver's several enzyme activities of de novo lipogenesis. The expression of these enzymes is regulated by the transcription factor SREBP1c [30]. All these processes cause the accumulation of hepatic TGs and the development of obesity. Later, obesity contributes to chronic inflammation via the stimulation of the nuclear factor-kappa B (NF-kB) pathway and produces inflammatory cytokines like interleukin (IL)-6 and tumor necrosis factor-alpha (TNF-α). An anti-inflammatory adipokine, adiponectin, is decreased in NAFLD, which further causes IR [8]. Moreover, FFAs, cytokines, and toll-like pattern recognition receptors (TLRs) also trigger molecular pathways contributing to IR. Additionally, the suppression of the nuclear receptor peroxisome proliferator-activated receptor alpha (PPAR-α) causes NAFLD by decreasing the FA β-oxidation in hepatocytes [2].

The second-hit hypothesis describes liver inflammation, excessive pro-inflammatory cytokines, mitochondrial dysfunction, oxidative stress, and fibrosis. The continuous activation of hepatic stellate cells and Kupffer cells and their apoptosis are the main contributors to hepatic fibrosis and cirrhosis [1]. Lipotoxic agents such as diacylglycerols (DAGs), lysophosphatidylcholine species (LPCs), and ceramides mostly follow the second hit theory and play a vital role in the pathogenesis of NASH [35].

Relation With Gut Microbiota

The intestinal microbiota (IM) consists of hundreds of species of four main bacterial phyla: *Firmicutes*, *Actinobacteria*, *Bacteroidetes*, and *Gammaproteobacteria*. IM plays a significant role in the pathogenesis of NAFLD by decreasing the tight junction protein expression. This results in increased intestinal permeability, which favors the transfer of bacterial endotoxin into the bloodstream [8]. This endotoxemia creates an inflammatory environment by producing pro-inflammatory cytokines, hepatic toll-like receptor 4 (TLR4), and plasma plasminogen activator inhibitor 1 expression that develops IR and hepatic lipid build-up. IM also ferments non-digestible carbohydrates into absorbable forms, which promote FA synthesis and reduce fasting-induced adipocyte factors in intestinal cells. This process inhibits the activity of lipoprotein lipase and drives the accumulation of TG in the adipose tissue [8].

Another mechanism involves inflammasomes consisting of nucleotide-binding domains (NLRPs) and leucine-rich repeat-containing proteins. The NLRP3 inflammasome activation is triggered by pathogen-associated molecular patterns (PAMPs) and damage-associated molecular pattern molecules (DAMPs)-mediated reactive oxygen species (ROS) production. A study demonstrated that inflammasome-mediated dysbiosis of IM leads to NAFLD advancement [8].

Consequences

Cardiac: NAFLD has more cardiovascular mortality than liver disease itself due to increased serum cholesterol, TGs, VLDL, and low-density lipoprotein [9]. NAFLD causes IR, dyslipidemia, hypertension, and increases procoagulant proinflammatory cytokines and subsequently results in the development of cardiovascular diseases. Ventricular hypertrophy, valvular calcification, and arrhythmias are some of the common cardiac complications of NAFLD, which mostly depend on the severity of the disease [9].

Diabetes mellitus: Patients with NAFLD have an increased risk of developing T2DM and vice versa [10,20]. This bidirectional relationship results from common pathophysiological causes such as obesity, IR, and metabolic syndrome [10]. NAFLD patients who have a normal initial diagnostic test for T2DM should repeat glycosylated hemoglobin testing at a minimum of a three-year interval [11]. In contrast, another study shows that screening for T2DM with glycosylated hemoglobin or an oral glucose tolerance test should be done annually [10].

Chronic kidney disease: There is a high risk of CKD in NAFLD due to the presence of some risk factors like T2DM, metabolic syndrome, obesity, and hypertension. The pathophysiology responsible for this phenomenon is perturbation of the IM, prothrombotic state, platelet activation, and increased uric acid levels [10]. Patients with NAFLD may present with a reduced estimated glomerular filtration rate (GFR) or albuminuria. Some experts have proposed screening strategies including yearly urine microalbumin, albumin/creatinine ratio, and GFR in patients with NAFLD for early detection of CKD, especially those with T2DM and obesity [10].

Inflammatory bowel disease: IBD with fatty liver is seen in patients who are older, diabetic, have a later disease onset, and have a greater BMI [13]. Many recent studies have shown that NAFLD is accountable for hepatic changes diagnosed in a patient with Crohn’s disease and ulcerative colitis, two main forms of IBD. There are some common pathogenic pathways such as inflammation, nutritional status, and genetic susceptibility that are believed to be responsible for the co-occurrence of IBD and NAFLD [13]. The shared pathogenetic pathway between IBD and NAFLD includes increased gut permeability by bacterial endotoxin and increased intestinal FA production, which subsequently worsens the ongoing chronic inflammation in the gut along with metabolic syndrome, obesity, and innate genetic susceptibility that have been gaining importance in favor of both ailments in recent years [13]. One systematic review from 2017 showed that certain immunosuppressive therapies used in the treatment of IBD can increase the level of hepatic aminotransferases, which can increase the prevalence of NAFLD [36]. The presence of NASH can restrict the use of some immunosuppressive agents due to the associated risk of drug-induced liver injury [36]. Though there are no precise recommendations for the management of NAFLD in patients with IBD, patients with IBD risk factors should undergo early screening, and, as a part of comprehensive management, both prevention and early treatment should be conducted efficiently [36].

Polycystic ovarian syndrome (PCOS): In comparison with healthy women, women with PCOS have a four times higher risk of developing NAFLD [12]. It is assumed that some common genes involved in the synthesis of androgens (CYP17, CYP11A, and SHBG gene), cytokines (IL-6, TNF-α, TNFR2) [12], and IR (insulin, INS-R) are responsible for the same disturbances in both PCOS and NAFLD [10,12]. NAFLD exacerbates IR in PCOS, which results in hyperandrogenemia, chronic anovulation, atherogenic dyslipidemia, and subclinical inflammation. IR also increases lipolysis and the flow of FFA to the liver and results in further hepatic fat accumulation in PCOS women [12]. Women with obesity and PCOS with IR have more chances of generating T2DM and impact liver function by developing liver cirrhosis and NAFLD [12]. Previous studies have recommended an increased threat of NAFLD in individuals with PCOS and involved androgen excess as an inherent operator [37]. Women who are diagnosed with PCOS-related androgen excess should have systematic NAFLD screening [37].

Other complications: Comorbidities such as OSA [10,20], neurocognitive dysfunction and depression [20], psoriasis [10], osteoporosis [20], and hypothyroidism [10,20] are also seen in patients with NAFLD.

NAFLD With Hepatocellular Cancer

About 40% of patients with NAFLD progress from NASH to cirrhosis to hepatocellular carcinoma (HCC), which is one of the causes of mortality in NAFLD. Whereas NAFLD is histologically characterized by isolated hepatic steatosis, NASH is defined by the presence of hepatic steatosis as well as acute and chronic lobular inflammation, hepatocyte ballooning, and zone 3 perisinusoidal fibrosis [15]. Although liver cirrhosis is not a prerequisite for HCC, patients with advanced fibrosis-cirrhosis are at an increased risk of HCC. NASH without cirrhosis can also progress to HCC [5,25]. Classic risk factors associated with NAFLD-HCC are older age, male population, and smoking. Moreover, fibrosis, obesity, diabetes mellitus, and hepatic iron accumulation are noteworthy risk factors [25]. HCC associated with NAFLD is mostly well differentiated and has a larger tumor size of 3-4 cm than other invasive phenotypes with hepatitis C virus infection [25,29]. In NAFLD with HCCs, solitary lesions are in 70-78% of cases. Compared to other chronic liver disease-induced HCC, NAFLD-HCC patients show lower levels of α-fetoprotein (AFP) [5]. Multiple signaling pathways associated with obesity, metabolic disorders, and NAFLD are responsible for NAFLD-HCC. TNF-α, IL-1, IL-6, and monocyte chemoattractant protein-1 (MCP-1), are up-regulated in the adipose tissues by adipocytes and macrophages. This phenomenon causes lipid accumulation and inflammation in the liver, eventually leading to NASH development and HCC progression. The PI3K/AKT/mTOR/PTEN pathway, mitochondrial dysfunction, and genetic alterations are also responsible for the progression of NAFLD with HCC, which is initiated by hyperinsulinemia and IR. Therefore, lifestyle intervention, insulin sensitizers, anti-inflammatory agents, anti-fibrosis, and chemopreventive agents can be preventive options for NAFLD-HCC [29].

NAFLD With Extrahepatic Cancer

NAFLD is not only associated with HCC but also with other extrahepatic cancers like colorectal cancer, intrahepatic cholangiocarcinoma, extrahepatic cholangiocarcinoma, uterine cancer, thyroid cancer, lung cancer, esophageal adenocarcinoma, as well as stomach cancer [10,11]. A higher serum aminotransferase in men with NAFLD is associated with a higher risk of thyroid cancer. NAFLD also increases the risk of lung and colon cancer in smokers. The risk of kidney cancer associated with NAFLD is also high in patients without T2DM [11]. Regardless of the origin, the chronic inflammation associated with NAFLD is believed to have a tumor-promoting effect on the body in the interplay of molecules such as NF-kB, signal transducer and activator of transcription 3 (STAT-3), and various proinflammatory cytokines, such as IL-1, IL-6, and tumor necrosis factor. The exact mechanism of cancer in NAFLD remains uncertain [11]. Although screening tests are not recommended for extrahepatic cancers, regular surveillance for HCC with US examinations should be done for individuals with NAFLD who have cirrhosis and portal hypertension [11].

NAFLD With COVID-19

**Figure 2:**
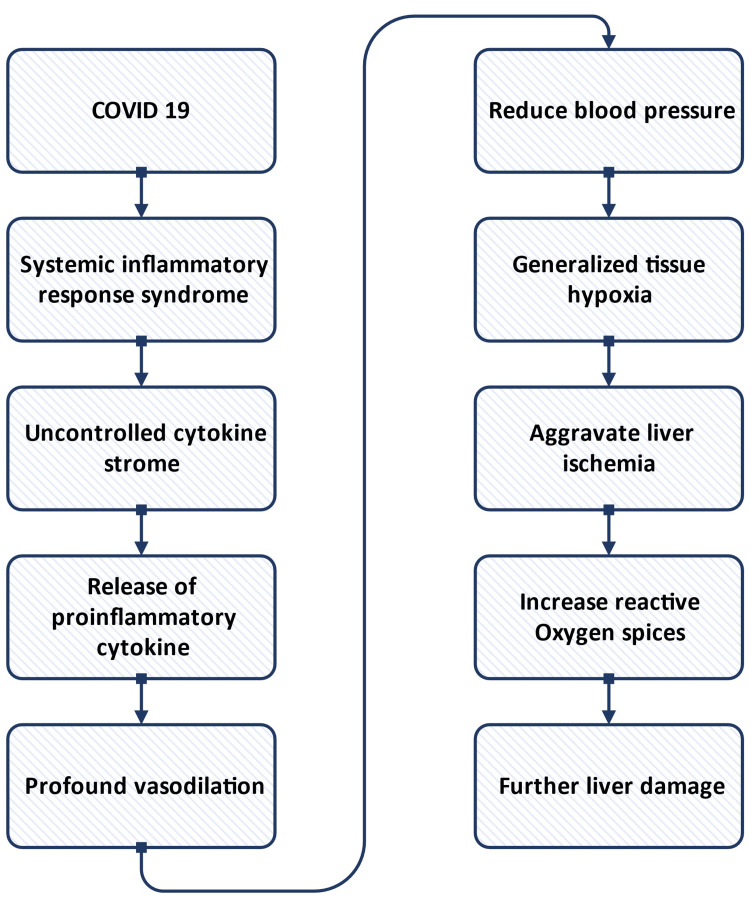
Mechanism of liver injury in COVID-19 patients. Image Credits: Mousumi Khanam

It has been reported that patients with pre-existing liver diseases like NAFLD are susceptible to developing severe COVID-19 [38]. Although COVID-19 mostly affects respiratory organs, it may present with extrapulmonary manifestations such as liver injury (incidence up to 53%) [38]. In COVID-19 patients, liver injury occurs due to dysregulated and uncontrolled systemic inflammation [38]. COVID-19 is a systemic inflammatory response syndrome that causes an uncontrolled cytokine storm that finally results in liver failure [38]. Figure 2 presents the mechanism of liver injury in COVID-19 patients. It is also suggested that COVID-19 has a direct viral effect on the liver and exacerbates pre-existing liver diseases.

NAFLD plays an important role in the outcome of COVID-19. It has been shown that during admission, patients who have pre-existing obesity and metabolic syndrome like NAFLD present with a severe form of COVID-19 [17].

During COVID-19 treatment, antiviral drugs like lopinavir/ritonavir, remdesivir, chloroquine, and tocilizumab can worsen liver dysfunction, especially in those who have underlying metabolic abnormalities like NAFLD [17]. A major focus should be given while treating COVID-19 patients with a history of NAFLD or other metabolic syndromes to rapid recovery [39]. It has been recommended that while treating COVID-19 patients, anti-inflammatory hepatoprotective drugs (like ammonium glycyrrhizate) should be used to accelerate recovery, and for hypoxic-ischemic injury, oxygen therapy is preferred [39].

Diagnosis and Investigations

In high-risk individuals with obesity (BMI > 30 kg/m^2^) or metabolic syndrome, the European guideline now supports the screening for NAFLD using US and/or liver tests. However, the AASLD continues to defend it, as there is no effective treatment [14]. Most of the time, NAFLD is asymptomatic and is only discovered by abnormal liver function or imaging test results during routine checkups. NAFLD might be suspected in patients who have persistently elevated serum aspartate aminotransferase (AST) and alanine aminotransferase (ALT), and fatty changes on US or CT scan [2]. However, NAFLD cannot be ruled out even if liver enzymes are within the reference range [21]. NAFLD is divided into NAFL and NASH based on histology. While NAFL and NASH both require the presence of >5% hepatic steatosis, NASH also requires signs of inflammation and hepatocyte injury. Fibrosis may not always be present in NASH [40]. But before confirming NAFLD, other causes of steatosis such as alcohol, viral hepatitis, parental nutrition, medications, starvation, inborn errors of metabolism, Wilson's disease, cholesteryl ester storage disease, fatty liver of pregnancy, and HELLP (H = hemolysis, EL = elevated liver enzymes, LP = low platelet count) syndrome must be carefully ruled out [2,3,30]. Additionally, NAFLD/NASH may potentially manifest after gastrointestinal surgery, such as an intestinal bypass or a pancreaticoduodenectomy [2].

Liver biopsy is the gold standard for the diagnosis of NAFLD. Along with assessing the presence and severity of liver fibrosis, it can also detect a distinction between the processes that are thought to be non- or slowly progressing (steatosis alone, NAFL) and patterns linked to the development of liver injury (steatohepatitis, NASH) [6,21]. Although it is intrusive, costly, and time-consuming, however, so far, there are no noninvasive techniques that are accurate enough to replace liver biopsy [41].

Hepatic steatosis is frequently detected via ultrasonography [21]. However, nonenhanced CT performs better than the US in determining the degree of fatty liver. For less radiation exposure, contrast-enhanced CT can be used, but it may be more appropriate for severe hepatic steatosis. Based on ultrasonic waves, controlled attenuation parameters (CAP) can distinguish between any degree of steatosis and no steatosis [18]. MRI, particularly using the proton density fat fraction (PDFF) technique, is the most precise noninvasive way to identify and measure hepatic steatosis [2]. Another highly successful MRI-based technique is magnetic resonance elastography (MRE), which uses a modified phase-contrast method to measure liver stiffness. In NAFLD patients, two-dimensional MRE is often used to evaluate liver fibrosis [18]. However, these imaging tests have their own drawbacks. US has poor sensitivity in patients with moderate steatosis (20%) and BMIs > 40 kg/m^2^. Also, it cannot detect the presence and severity of inflammation (NASH) and fibrosis [21]. The downsides of CT include radiation exposure, cost, Hounsfield unit (HU) fluctuation depending on device setup, and inaccuracy from concomitant iron/copper depositions [2]. CAP's main drawbacks are operator dependence and inadequate sensitivity for moderate steatosis [18]. As MRI is expensive equipment, many basic care clinics and other facilities cannot afford it [2]. MRE cannot be used on patients with excessive hepatic iron deposition [18].

Usually, biomarker panels cannot differentiate between mild and moderate steatosis. Most studies are utilizing biomarker panels for diagnosing NAFLD, based on subpar gold standards with US or proton magnetic resonance spectroscopy (1H-MRS) [18]. The fatty liver index (FLI) calculates BMI, waist circumference, TGs, and gamma-glutamyl transferase, and the total score ranges from 0 to 100. However, it fails to identify patients with severe illnesses (NASH or advanced fibrosis) [18,21]. The NAFL screening score is a simple model that calculates age, fasting blood sugar, BMI, TGs, ALT/AST, and uric acid to detect NAFLD [18]. Age, AST and ALT levels, and platelet count are used to calculate the fibrosis-4 (FIB-4) score, which can be used to distinguish patients who may have advanced fibrosis [21]. A recent study showed that NFS and FIB-4 scores were as excellent as MRE and more precise than other clinical scores to predict advanced fibrosis in patients with NAFLD [30]. However, due to a high probability of false positives, a low threshold of 0.12 for identifying fibrosis is advised for the elderly [18].

The NASH test combines 13 factors to diagnose NASH and categorizes it into three groups, namely, NASH, borderline NASH, and no NASH [18]. The NASH Clin score, which used to include AST, fasting insulin, and the PNPLA3 genotype at rs738409, was renamed to the "NASH ClinLipMet score" by Zhou et al. after metabolic syndrome-based components were included to increase the accuracy of diagnosing NASH. However, it is expensive and difficult to do in clinical practice, so it is better suited for research [18]. However, some other diagnostic performance enhancements, including Fibroscan and enhanced liver fibrosis (ELF), showed remarkable advancements in tracking disease progression and treatment response [21].

Though the mechanism is not clear, the features of the gut microbiota may act as diagnostic or prognostic markers. A collection of microbial markers developed from metagenomic sequencing, along with age and BMI, can accurately identify patients with NAFLD who have advanced fibrosis [19].

Treatment

Diabetes should be screened in newly diagnosed NAFLD patients [20]. The patients should be screened for diabetes every one to two years even though they do not currently have the disease because they are at a higher risk of developing incident diabetes [20]. Metformin, a treatment option of choice for T2DM, has been shown to lessen steatosis in individuals with NASH and to avoid liver events in T2DM patients with advanced NASH [21]. Liraglutide has been shown in studies to have a favorable impact on NASH and the development of fibrosis by lowering body weight, even in patients who do not have T2DM [42]. The thiazolidinediones group, which includes the peroxisome proliferator-activated receptor (PPAR) agonists rosiglitazone and pioglitazone, has anti-steatotic, anti-inflammatory, and to a lesser extent, therapeutic benefits in NAFLD [42]. In a phase 2 study, glucagon-like peptide-1 receptor agonists (GLP-1 RAs) showed improvement in liver histology in individuals with NASH [20], in addition to improvement in cardiovascular and all-cause mortality in high-risk T2DM patients. The development of HCC connected to NAFLD is prevented by statins [5]. Statins have a variety of actions, including those that are antiproliferative, proapoptotic, immune-modulatory, antiangiogenic, and anti-infective [5]. Statins block the post-translational modification (prenylation of small G proteins) of the RAS/Rho superfamily by preventing the conversion of 3-hydroxy-3-methylglutaryl coenzyme A (HMG-CoA) to mevalonate, a precursor to cholesterol [5]. The hepatocarcinogenesis process stage of Myc activation is likewise inhibited by atorvastatin, resulting in HCC regression [5]. Statins’ proapoptotic effects were demonstrated to be caused by their engagement in the RAF/MEK-ERK pathway, which they did by activating caspases and lowering Bcl-2 [5].

Bariatric surgery has gained acceptance for obese patients with a BMI of >40 kg/m2 and >35 kg/m2 with comorbidities according to the recommendations by the National Institute of Health (NIH) [40]. It improves the metabolic function of lipid metabolism and inflammatory pathways, which are associated with the pathophysiology of NAFLD. One recent meta-analysis [40], which included 21 studies of Roux-en-Y gastric bypass, adjustable gastric banding, sleeve gastroplasty, vertical gastroplasty, and multiple procedures with a total of 2374 patients, reported improvement in steatosis. We need further studies to investigate whether bariatric surgery has a potential cardioprotective effect in the setting of NAFLD or NASH.

The primary therapy for NAFLD is lifestyle changes, and these changes may be more difficult to make than other treatment options, which begin with a reduction in red meat, trans fat, processed carbs, and high-fructose corn syrup intake, as well as foods that are deficient in fiber and high in calorie density. A calorie deficit of 500-1000 calories per day is also included, as well as an increase in moderate-intensity exercise [28]. The Mediterranean diet, which is often strong in monounsaturated fatty acids (MUFAs) and omega-3 FA and avoids red meat, processed foods, processed meat, and refined sweets, is another dietary intervention that has been studied. Ryan et al. investigated how, compared to the control diet, the Mediterranean diet resulted in a higher reduction in hepatic steatosis by enhancing insulin sensitivity [43].

In a few studies, the TNF-α inhibitor pentoxifylline improved IR and liver enzymes. However, larger studies are required to fully assess the drug's benefits [34]. Obeticholic acid, a farnesoid X receptor agonist recently licensed by the FDA for the treatment of primary biliary cholangitis, has improved steatohepatitis and fibrosis during the course of a large, phase II clinical trial [34]. In a phase II trial, the dual PPAR-alpha/gamma agonist elafibranor has shown benefits in histologic NASH markers, including stabilization of fibrosis [34].

Prevention

The complex nature of NAFLD pathophysiology makes the development of universal preventative strategies difficult. For NAFLD, screening, which is the most essential preventative technique for detecting those at risk, is not yet clearly characterized. Screening is now utilized for people who are at risk of developing HCC. However, it is not yet used in clinical practice for those who are at risk of developing NAFLD. The use of US and liver function blood tests can be quite beneficial in this regard. Transient elastography (TE) has also been recommended as a community-based screening technique for certain subgroups of people [44]. Patients with obstructive sleep apnea are another category of patients who are thought to be at a higher risk of developing NAFLD, NASH, or fibrosis, and screening of these patients may be beneficial [44]. Furthermore, considering the importance of PNPLA3 polymorphisms in the etiology of NAFLD and, more crucially, HCC, genetic testing of particular groups, such as individuals with NAFLD but no metabolic syndrome symptoms, may be prudent [45].

Limitations

In this study, we excluded manuscripts that were published before 2017. We also eliminated studies related to the pediatric population and animals. Due to the limited studies published, there is no strong evidence that COVID-19 has a significant role in the development of NAFLD. On the contrary, it is a matter of dispute that patients who are suffering from NAFLD have vigorous COVID-19 consequences [38]. Moreover, in our study, we considered biopsy as a gold standard for staging NASH and fibrosis. However, biopsy has some limitations, including sampling error, inter-rater reliability, cost, and acceptability for long-term observation [27].

## Conclusions

NAFLD is a global health problem. Current diet and lifestyle trends are increasing the risk of obesity and T2DM, which are ultimately increasing the incidence of NAFLD. This systematic review analyzes the incidence and prevalence of NAFLD among various populations as well as the relationship of NAFLD with different variables. Global awareness campaigns are needed to enhance knowledge of NAFLD and the ailments it is linked to, allowing global actions to alter the disease's progression. Though USG and MRI are used to diagnose NAFLD, further study is required to establish noninvasive biomarkers, which will not only diagnose NAFLD but will also differentiate among different stages of disease progression. Searching for the perfect NAFLD treatment is hindered by its complexity and heterogeneity, so current treatments are often focused on improving one of the hallmark factors of NAFLD (steatosis, inflammation, and fibrosis). As genetic, lipidomic, and metabolomic information grows, the future seems bright for tailoring treatments for NAFLD. In the future, early detection will allow for quick single or combined therapy with top-of-the-line medications that have been tuned for optimum benefit and minimal side effects. The development of tailored medicines to stop the progression of liver disease into advanced stages is the focus of current research, particularly in NAFLD.
